# Comparison of Two Different Morphological Methods to Study the Pronotum of Cimicidae: Bed Bugs, Bat Bugs, and Swallow Bugs

**DOI:** 10.3390/insects13121155

**Published:** 2022-12-14

**Authors:** Dora M. Rajonhson, Chadchalerm Raksakoon, Anon Payakkapol, Sébastien Dujardin, Jean-Pierre Dujardin, Rutcharin Potiwat

**Affiliations:** 1 Department of Medical Entomology, Faculty of Tropical Medicine, Mahidol University, 420/6 Ratchawithi Road, Ratchathewi, Bangkok 10400, Thailand; 2 Department of Chemistry, Faculty of Science, Kasetsart University, Bangkok 10900, Thailand; 3 Institut de Recherche pour le Développement (IRD), UMR INTERTRYP IRD-CIRAD, University of Montpellier, F-34398 Montpellier, France

**Keywords:** Cimicidae, ectoparasite, outline-based morphometry, pronotum, linear morphometry

## Abstract

**Simple Summary:**

Several members of the Cimicidae family are ectoparasites of vertebrates. Some of these are economically and medically important, e.g., the bed bugs, which are a global pest affecting humans. The development of a method to easily identify species of the Cimicidae family, despite the high morphological similarity of its members, will bring benefits such as the early detection of emerging infestations, facilitating the setup of adequate control and management measures. One of the existing methods, traditional morphometry (linear measurements and ratios of an object), is a demanding process involving the examination of many morphological features of well-preserved samples. An alternative approach that uses only one morphological feature could be an asset. Therefore, we assessed the use of a single organ, the pronotum, to classify Cimicidae via two methods: traditional and geometric morphometry (a method capturing the geometry of an object using coordinates as opposed to simple linear measurements and ratios). Both methods were effective in classifying members of the family based on the pronotum; however, better quality data were obtained with geometric morphometry. We recommend adopting the latter approach in future surveillance programs for medically important members and poultry pests of the family.

**Abstract:**

An infestation of a Cimicidae (Hemiptera: Cimicidae) member, especially the bed bug, can cause economic loss and impact health. A cost-effective and user-friendly method for identifying the infesting species will help with the early detection and control of infestations. A linear morphometric method is often used, but it requires the examination of many characters and a highly preserved specimen. We conducted a comparative morphometric study of the effectiveness of Cimicidae classification using a single organ, the pronotum, through outline-based and linear morphometric methods. Bat (*Stricticimex parvus*), human (*Cimex hemipterus*), and bird (*Paracimex* sp.) ectoparasites were subject of the study. With both methods, the properties of size and shape were compared and used separately to classify the specimens. Classification analyses of the two methods provided similar results, but more informative variables of size and shape were obtained with the outline-based approach. Size, as analyzed with the outline-based method, could detect sexual dimorphism, and produced better reclassification. The shape variables obtained from the linear measurements were strongly influenced by size variation, much more than the ones obtained from coordinates describing the pronotum contours. Our data suggest that the outline-based approach provides better characterization variables, thus we recommend them for a wider use in other Cimicidae family members.

## 1. Introduction

Members of the Cimicidae family (Hemiptera: Cimicidae) are hematophagous ectoparasites of medical and veterinary importance. The involvement of Cimicidae in the transmission of human disease has been a concern and focus of scientific investigations for more than 100 years [1,2]. Most studies have assessed the potential role of either the cosmopolitan bed bug *Cimex lectularius* (Linnaeus) or the tropical and subtropical bed bug *Cimex hemipterus* (Fabricius) in human disease transmission. Despite the thorough investigations performed to date, the epidemiological roles of Cimicidae members as infectious disease vectors remain unclear [3], however, additional aspects for their incrimination are yet to be addressed, such as their physiology/ecology and use of cutting-edge approaches [2]. Bed bugs infestation is likely to cause illnesses [3], including behavioral, morphological, physiological distress, and economic loss [4]. More than 45 human pathogens distributed in several categories including bacteria, protozoa, helminths, and viruses were found in bed bugs [5]. Similarly, two bat bugs, *Cimex insuetus* (Ueshima) and *Stricticimex parvus* (Ueshima), were suspected of carrying and transmitting zoonotic virus to humans [6]. Additionally, the swallow bug, *Oeciacus vicarius* (Horváth), was reported to invade human interfaces and bite people [7].

Widespread bed bug eradication campaigns took place between the early 20th century and the end of the Second World War. Therefore, with their recent re-emergence, there is an overall lack of knowledge about their appearance across generations and among populations [8]. Moreover, similarities in general appearance among Cimicidae members can make the morphological identification as well as clarifying the species status of some members difficult [9,10,11]. The ability to identify Cimicidae members will bring benefits such as the early detection of emerging infestations, facilitating the setup of adequate control and management strategies. A cost-effective and user-friendly method that allows the identification of these species will therefore help with the early detection and control of infestations.

Through host adaptation, members of the Cimicidae family have developed major morphological differences: (i) longer legs allow for more active dispersion of the species amongst their hosts; (ii) changes to the widths and lengths of rostral segments are a response to different host skins; (iii) differences in the dimensions of the antennal segments or eyes aid with host-seeking activities; and (iv) the presence of hairs correlates to the type of body surface on the host species [12,13,14]. As a result, the ectoparasite family is divided according to their host specialization in several vertebrate classes as the species investigated in this study: bats (*Stricticimex parvus*), humans (*Cimex hemipterus*), and birds (*Paracimex* sp. (Kiritshenko)). Some members of the family can also feed on other warm-blooded animals other than their main host, such as the bed bugs [15]. To date, the family contains 110 described species within 24 genera [16], 12 of which are ectoparasites of bats, 9 are associated with birds, and 3 are related to humans [12]. Despite the above host-driven modifications, discriminating between species and genera is still challenging [10,12,13].

Morphological features have traditionally been used to identify species in the Cimicidae family, and accurate classification calls for the use of internal characteristics, demanding fresh specimens and expertise in insect dissection [12]. Indeed, the unique mating behavior of the family, i.e., traumatic insemination [17], has instigated a high level of diversity in the female paragenital system; not only is its internal organization adequate to classify most genera but it can also be used to demonstrate the evolution of Cimicidae family members [12]. However, the dissection of the paragenital system is difficult to implement in routine taxonomic work.

Before the introduction of molecular methods, Cimicidae family phylogenetic relationships were assessed via morphological differences (often those related to the reproductive organs), crossbreeding experiments, the numbers of chromosomes, and host associations [10,12,18]. Recent sequencing of family members’ mitochondrial cytochrome oxidase subunit I (cox1), 16S rDNA, internal transcribed spacer 2 (ITS2), and/or 18S and 28S rRNA genes allowed phylogeny to be investigated based on the molecular data [13,19,20,21]. The molecular approach has supported the traditional division of groups, species, and genera of the family as well as proposing restructuration in some instances. *Oeciacus* spp. and *Cimex* spp. are traditionally considered as separate genera based on morphological differences [12]. Following molecular analysis, change in taxonomy was suggested since the two species were found to be paraphyletic [22]. Their morphological differences are much more closely correlated to their host association (bird- and bat-associated species, respectively), than to their genetic differences, which is also the case for the *Cimex pipistrelli* group (Linnaeus) [23]. The molecular approach has greater specificity and sensitivity because it can be applied at any life stage of the species and does not require morphologically well-preserved specimens. However, it requires expertise, and it is time-consuming and relatively expensive.

A matrix-assisted laser desorption/ionization time-of-flight mass spectrometry (MALDI-TOF--MS) method was recently developed to distinguish the bed bug species *C. lectularius* and *C. hemipterus*, the most common members of the Cimicidae family, which have a worldwide occurrence and a preference for human environments [24]. Despite the effectiveness of the method in differentiating between the two bed bugs to the species level, the necessity for an MS device can be a major obstacle. As for the running cost, it is left to each laboratory to make the methods available for routine species identification on a case-by-case basis. The expenses involved in MALDI-TOF MS are less restrictive than those for molecular techniques [25].

As such, the currently available classification systems have limitations and investigating other techniques for the description and nomenclature of family phylogeny could lead to a valuable alternative approach. Morphometry, which measures the size and, separately, the shape of organisms offers interesting possibilities. The use of this method in the Cimicidae family traces back to 1966, when Usinger [12] used linear measurements between anatomical points, mainly for systematics at the species level [11]. For instance, to differentiate between *Cacodmus villosus* (Stål) and *Cacodmus sparsilis* (Rothschild), various characters belonging to the thorax and genital segments were measured [26]. Simple measurements of length, width, or ratios of characters were used. Recently, a morphometric analysis of members of the Cimicidae family required the measurement of up to 61 characters, which for the shape analysis were subsequently reduced by eliminating characters that are correlated with body size and dimension [23]. However, it may be possible to use only a few characters, or ideally one, to distinguish or recognize important species. In linear morphometry, the pronotum width, a precisely measurable characteristic, was found to be highly correlated with the overall body size of Cimicidae members [12]. In terms of shape, the visual aspect of pronotum morphology has been used on multiple occasions to describe differences between species [11,12], e.g., *C. hemipterus* and *C. lectularius*, in which the pronotum is characterized by narrow or broad lateral lobes, respectively [12,27]. Despite existing statistical methods designed to extract shape information from linear measurements [28], no such attempt has been published for Cimicidae. We present here the use of the Darroch and Mosimann method to distinguish three Cimicidae species [29].

We compared the linear measurement method with the geometric morphometric one. Geometric morphometry has revolutionized the linear morphometric approach [30] and was successfully applied to the field of taxonomy in medical entomology [31,32]. Two main geometric methods exist, and both are based on spatial coordinates describing either closed curves (pseudolandmarks) or specific anatomical points (landmarks). The landmark approach was recently used to describe the morphological difference within several populations of the tropical bed bug, *C. hemipterus* [33]. Population segregation was observed because of a general body shape change, including the pronotum [33]. However, segregation related to the pronotum alone was not assessed. The pronotum is an important taxonomic character in Cimicidae. Studying its shape, for species differentiation at inter- and intraspecific levels, could be a valuable tool to study phenotypic variation, local adaptations, and population divergence [34]. The outline-based approach has not been explored in Cimicidae; it allows a direct description of any feature, for instance, the contour of the pronotum, and does not need a recognition of specific points from one individual to another. Both geometric methods produce separate analyses of size and shape, with the additional possibility of visualizing shape changes among groups.

Hence, in this study, we used one anatomical structure, the pronotum, as a possible taxonomic character to compare two approaches: (i) linear morphometry and (ii) outline-based morphometry. By exploring the possible taxonomical signal of the pronotum shape, our long-term objective was to provide a cost-effective identification tool for the Cimicidae family.

## 2. Materials and Methods

### 2.1. Specimen Collections

The ectoparasites collected have distinct hosts: human, bird, and bat. They were collected from their respective host living areas across three different sites in Thailand and preserved in 70% ethanol. Information regarding sex, sampling size, year of collection, and collection areas of the ectoparasites is summarized in Supplementary Table S1. Bed bug samples were collected from Bangkok Province, Thailand and maintained at the Department of Medical Entomology, Faculty of Tropical Medicine, Mahidol University since 2014. Swallow parasite samples were collected by Chittsamart et al., 2015 [35] from the nests of edible-nest swiftlets (*Collocalia fuciphaga*, Apodiformes: Apodidae, Thunberg) in Lang Ga Jiew, a small offshore island close to Chumphon Province, Thailand. Bat parasites were from a bat limestone cave located in Thap Kwang, Kaeng Khoi, Saraburi Province, Thailand.

Twenty-six samples of each species were mounted for morphological identification and photographed for morphometric characterization. Morphological identification was conducted under a stereomicroscope following the identification keys of Usinger [12] and other keys. According to the Usinger’s keys [12], the human ectoparasites, commonly called bed bugs, were identified as *C. hemipterus* and the swallow ectoparasites belonged to the genus *Paracimex* sp. Using Ueshima’s identification keys [36], the bat ectoparasites were identified as *S. parvus*.

### 2.2. Specimen Mounting, Photography, and Morphological Identification

Before morphological identification, the specimens were mounted between glass and cover slides using Hoyer’s medium as a rapid clearing semi-permanent mounting medium [37]. The medium was composed of distilled water (50 mL), Arabic gum (acacia powder) (a stabilizer, emulsifier, and thickening agent) (30–40 g), chloral-hydrate (200 g), and glycerin (a moisturizer to treat or prevent desiccation) (20 mL). The ingredients were mixed in the above order at room temperature, then left to stand for several hours to allow any bubbles to disappear from the solution before storing in an airtight bottle. To assess the clearing effect of the mounting medium, the specimens were photographed from day 1 to day 5 post-mounting. Daily observations of the mounted specimens were conducted during that period. Specimens were found to be the clearest on day 5; consequently, pictures taken on the 5th day post-mounting were used for morphometric analysis. The clearest images were evaluated by eye.

**Figure 1:**
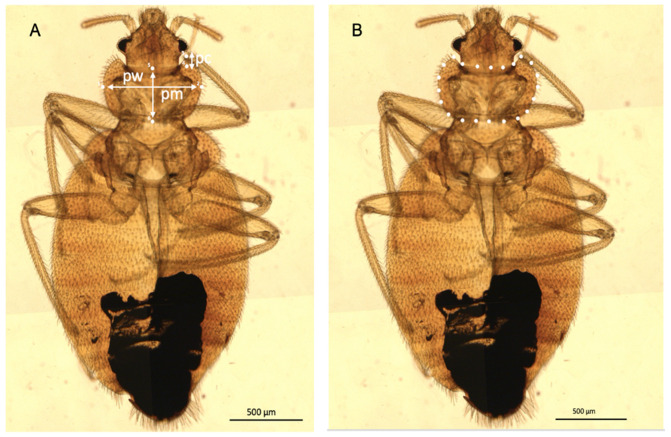
(**A**) Dorsal view of *Cimex hemipterus* male taken with the Zeiss Axio Imager M2 with the scale 0.5 mm. Illustration of the linear measurements applied to the pronotum and used as input for the linear morphometry. pw, pronotum width; pm, pronotum length (medial); pc, anterior pronotal concavity depth. (**B**) Picture illustrating the pseudolandmarks (white dots) digitized along the pronotum contour and used as input for the outline-based morphometry.

Images were taken with the Zeiss Axio Imager M2 (Zeiss, Oberkochen, Germany), an upright, fully motorized, PC-controlled, high-performance research microscope. To enhance the focus of the images, the software autofocus was used with a defined tile region. The images were combined and arranged in a form of a grid to produce a sharper image. In each picture, the scale has been converted from pixels to micrometers (Figure 1).

### 2.3. Linear Morphometric Analysis

Three linear measurements of the pronotum (Table S2) were recorded for the mounted specimens: pronotum width (pw), pronotum length (pm), and anterior pronotal concavity depth (pc) (Figure 1). These measurements were taken using an ocular micrometer (4X objective, NA 0.10, 10X ocular lens) (Olympus, Tokyo, Japan) (Table S2, Figure 1).

The statistical analyses (Table S2) followed the approach of Darroch and Mosimann [29]. Thus, for males and females, the average of the three log-transformed variables (pw, pm, and pc) represented estimates of their global size (hereafter named “log-size”). The log-transformed measurements were centered and subjected to a principal component analysis (PCA). Due to the loss of one degree of freedom (due to centering), the last principal component (PC) was removed for subsequent analyses. The two remaining PCs, called log-shape ratios (LSR1 and LSR2), represented the shape variation [29]. The differences in pronotum size (Table 1) were investigated by non-parametric ANOVA based on permutations between groups (1000 cycles) [38].

### 2.4. Outline-Based Morphometrics

Using the images taken with the Zeiss Axio Imager M2 (Zeiss, Oberkochen, Germany), the external contour of the pronotum (Figure 1) was manually digitized using XYOM software, https://xyom.io/, accessed on 8 April 2022 [39].

The raw coordinates (pseudolandmarks) obtained from males and females were submitted to elliptic Fourier analysis (EFA) separately. This analysis produced variables describing (i) the pronotum shape (normalized elliptic Fourier (NEF) coefficients) and (ii) the global pronotum size, as estimated by the semi-major axis of the first ellipse [40].

To analyze differences in pronotum size (Table 1) among the ectoparasites, non-parametric ANOVA with 1000 permutations of the semi-major axis of the first ellipse was performed, and *p*-values were obtained [38].

### 2.5. Clustering and Reclassification

Reclassification based on size obtained from either the linear or outline-based morphometric data was performed using the maximum likelihood approach [41].

For shape, either LSR or NEF, both supervised and unsupervised classification were performed. A supervised classification makes use of the labels assigned to the data (here the ones given by the morphological determination). On the contrary, an unsupervised classification looks for natural grouping of individuals without using their labels.

Unsupervised classification used the classical hierarchical agglomerative algorithm (HAC) illustrated by an UPGMA tree based on Euclidean distances between shape variables. Supervised classification was performed as a validated one, i.e., each reclassified case did not contribute to the model used to perform the classification. The model was an artificial neural network (ANN) making use of a simple multilayer perceptron (MLP) with a back-propagation algorithm [42]. The method has been recently applied to morphometric data, including outline-based morphometrics [43,44]. Following a process of trial-and-error, a single (hidden) layer composed of three neurons provided the best results. We used as input the total number of variables instead of a subset of their PCs. All specimens were separately classified 10 times, then an average classification score and its standard error were computed. The “accuracy” (Table 2) was the percentage of individuals correctly identified at the end of the procedure and is provided with the standard deviation.

### 2.6. Allometry

The influence of size variation on the shape variables of males and in females (the allometric effect) was estimated through the determination coefficient by regressing the shape variables onto the estimate of size. For the linear measurement method, the LSR1 and LSR2 were regressed onto the log-size; for the outline method, only the two first PCs of NEF coefficients were regressed onto the semi-major axis of the first ellipse.

### 2.7. Software

For specialized statistical morphometric analyses [29] and contour digitization, we used the free online software XYOM [39]. To perform the ANN-based validated classification, we used the multilayer perceptron program written in JavaScript, which is available at https://www.npmjs.com/package/mlp, accessed on 8 April 2022.

## 3. Results

### 3.1. Size Variation of the Ectoparasites

**Figure 2:**
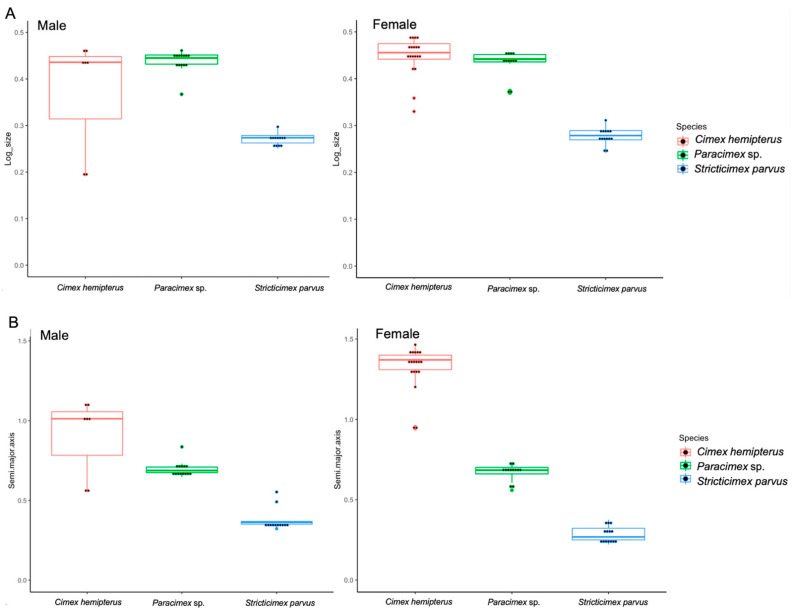
Quantile boxes showing the relative variation in the global size of the pronotum. Variations in size among males (left) and females (right) were derived via (**A**) the linear approach (log-size) and (**B**) outline-based morphometry (semi-major axis of the first ellipse). Boxes show group medians that separate the 25th and 75th percentiles.

For each ectoparasite, and for each sex, the global size of the pronotum was assessed using the linear and outline-based techniques. The two methods did not converge exactly to the same relative patterns of size (Table 1, Figure 2).

According to linear morphometry, *C. hemipterus* and *Paracimex* sp. did not have any significant difference in pronotum size, but they both had a significantly larger pronota than *S. parvus* (*C. hemipterus* male test, df = 32, *p* = 0.02; *Paracimex* sp. male test, df = 32, *p* = 0.001; and for both *C. hemipterus* and *Paracimex* sp. females test, df = 44, *p* = 0.001) (Table 1). No significant difference in pronotum size was observed between males and females, apart from in *C. hemipterus*, where a significant sexual dimorphism was observed, with females having larger pronota than males (*C. hemipterus* male and female test, df = 25, *p* = 0.03).

In contrast to the global pronotum size estimated from linear measurements, the estimate extracted from the outline analysis (the semi-major axis of the first ellipse) disclosed significant (*Paracimex* sp. and *C. hemipterus* females test, df = 44, *p* < 0.001) or nearly significant (*Paracimex* sp. and *C. hemipterus* males test, df = 32, *p* = 0.06) differences between *Paracimex* sp. and *C. hemipterus*. When we examined the global size of the *S. parvus* pronotum, in agreement with the linear morphometrics, it remained significantly smaller than that of *Paracimex* sp. (male test, df = 32, *p* = 0.001; female test, df = 44, *p* = 0.02) and *C. hemipterus* (male test, df = 32, *p* < 0.001; female test, df = 44, *p* < 0.001) (Table 1). Moreover, sexual dimorphism in pronotum size was observed for *C. hemipterus*, with females having larger pronota than males (*C. hemipterus* male and female test, df = 25, *p* < 0.001), and in *S. parvus*, with males having larger pronota than females (*S. parvus* male and female test, df = 25, *p* < 0.001). Shape Variation of Ectoparasites

**Figure 3:**
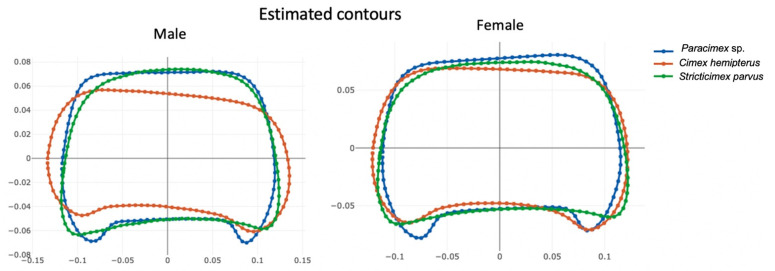
Group means of the ectoparasite outlines, with X and Y axes as reconstructed coordinates after inverse Fourier function.

For both males and females, visualization of the pronotum shape derived from the outline analysis revealed three distinct contours (Figure 3).

*C. hemipterus* showed an anterior margin that was slightly excavated compared with *S. parvus*, while the extremity margin of *Paracimex* sp. was more elongated than that of the two other species.

### 3.2. Unsupervised Classification

**Figure 4:**
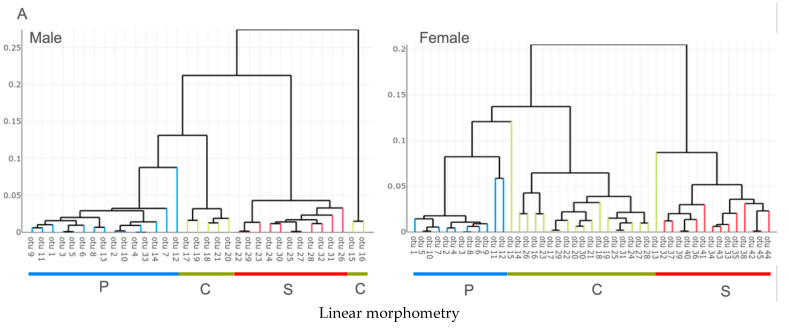

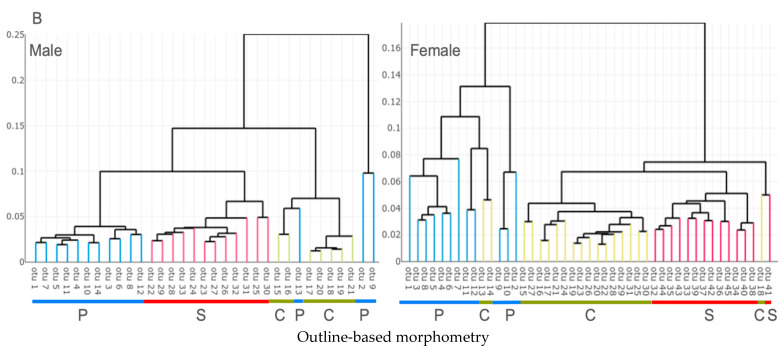
Shape-based hierarchical agglomerative clustering analysis derived from either log-shape ratios (**A**) or normalized elliptic Fourier coefficients (**B**) between males and females. Individuals are plotted on the horizontal axis as operational taxonomic units (OTU). Capital letters P, C, and S indicate the affiliated species, as to *Paracimex* sp. (P), *Cimex hemipterus* (C), and *Stricticimex parvus* (S).

In linear morphometry, both the male and female HAC clustered into three groups in accordance with their respective species. Female *Cimex* sp. were subdivided into two separate clusters (Figure 4A), and two individuals related to either the *Paracimex* or *Stricticimex* cluster (Figure 4A). Male specimens also included two apparently outlier individuals, located externally of the global tree (Figure 4A). Thus, using the linear measurement-derived shapes as criteria for clustering, HAC clustered most male (94%) (31/33) and female (95%) (43/45) specimens in accordance with our a priori morphological determination.

In outline-based morphometry, the formation of three groups that corresponded to their respective species was also prominent in both males and females (Figure 4B). However, in males, the *Paracimex* group was split into two separated clusters, plus one individual that was found in the *Cimex* cluster (Figure 4B). Some female *Cimex* were found either in the *Paracimex* (two individuals) or the *Stricticimex* (one individual) clusters (Figure 4B). Thus, the unsupervised classification using the outline-based approach clustered 91% (30/33) of males and 93% (42/45) of females in agreement with the pre-established groups.

### 3.3. Supervised Classification

A high percentage of individuals, both males and females, were correctly assigned to the relevant species using either the linear or outline-based morphometry (Table 2).

Using size only as a criterion for classification, with the linear approach, the specimens were correctly assigned for 61% of males and 71% of females. Using the outline-based approach, it correctly classified 82% of females and 85% of males (Table 2).

The MLP-based validated reclassification using shape only (LSR, NEF) scored highly for correct assignments whatever the morphometric approach, ranging from 94% to 99% (Table 2).

### 3.4. Allometry

In both sexes, the log-size (linear morphometry) was highly correlated with the first log-shape ratio (determination coefficient > 91% in both sexes, Table 3).

The equivalent study performed on geometric variables did not reach such a high percentage in terms of the size contribution to shape variation (from 2% to 15%, Table 3).

## 4. Discussion

Although morphological identification involving linear morphometry remains the gold-standard way to identify species of the Cimicidae family, it requires a well-preserved sample on which all characters needed for the identification remain and are free from any damage. The characters (bristles, antennae, and legs) commonly used in Cimicidae taxonomy can be easily and frequently broken off during sample preparation and transportation. For damaged samples, morphological identification becomes challenging and could be misleading. This is one of the best reasons to test the usefulness of a single anatomical feature that is not prone to damage with a method capable of extracting its taxonomic properties. We selected the pronotum because it is not prone to damage, and its visual aspect is already listed among the criteria suggested for morphological identification of Cimicidae, for instance, *C. lectularius* and *C. hemipterus* [12]. The pronotum is an easily identifiable feature that is well defined and separated from the rest of the thorax. Moreover, through several observations, the pronotum aspect was not listed among the morphological features that change within a species across different hosts [10,12,23], which again makes it a promising taxonomic character.

The unsupervised classification presented here had the objective of evaluating the taxonomic signals embedded in the shape variables generated by the different approaches. A simple visual examination of the resulting classification trees (Figure 4) strongly suggested that the pronotum is an informative character.

The validated reclassification presented here had the objective of assessing how our results could be generalized to an independent dataset. Each individual was iteratively removed, and its assignment computed following analysis of the remaining data (the “leave-one-out” method, also known as “validated reclassification”). Due to the small number of samples, we did not apply canonical variate analysis (or linear discriminant analysis), but instead selected the ANN method, which is not restricted to linear models and does not impose the austere assumptions related to canonical variate analysis [45]. This machine learning method may require a lot of data to achieve an accurate level of performance [46]; however, the scores obtained here were satisfactory as they ranged from 94% to 99%, whatever the morphometric approach.

We showed that the advantage of using the outline-based approach instead of the linear one is because of the quality of both size and shape estimators (Figure 2 and Figure 4) rather than the classification scores.

The shape variables computed from the linear measurements (LSR) and shape variables resulting from outline-based analysis (NEF) had different degrees of quality. The former showed a very high degree of size influence that could not be removed by the multivariate method used [29] (Table 3). These allometric residues, although still present, were much less frequent in the shape variables produced by the outline-based approach (Table 3). Due to the relatively low allometric residue occurrence of geometric shapes (Table 3), the resulting classification scores promise more stability. Not only are shapes more informative when derived from the outline method, the size is also. The global size estimate based on the outline-based approach allowed for a higher reclassification accuracy (82–85%) than the one based on linear measurements (61–71%) (Table 2). It was also able to detect the sexual dimorphism both in *C. hemipterus* and *S. parvus* pronotum size as opposed to the only detection in *C. hemipterus* with the linear method (Table 1). Thus, the outline-based analysis provided a more informative estimate of size and more independent shape variables, and its raw data were less laborious to capture. Moreover, contrary to the linear method, the outline-based approach allowed for a visual reconstruction of pronotal shape variation (Figure 3) [30].

Although the measurements conducted on the pronotum were applicable for genus and species distinction, including the genus *Cimex* spp. [12,13,23,47], the low number of linear measurements used (Table S2) could explain the relatively low quality of the shape variables. Some authors have recommended using a much larger panel of measurements [13,23]. Additional linear measurements of other features of the pronotum are likely to yield better estimates of global size and shape. This requirement makes the linear measurements approach a relatively time-consuming one.

Contrary to the recent landmark-based approach on Cimicidae [33], our geometric approach was restricted to the outline technique, excluding the landmark-based one, because of our difficulty in identifying valid landmarks on the pronotum (data not shown). In our study, in the absence of type I landmarks, and the small number of possible type II landmarks [48], we turned to the use of the shape outline, which was based on the digitization of pseudolandmarks [49]. The multivariate nature of outline-based morphometric analyses may require large sample sizes for each species. Such large samples may be difficult to obtain from the field when a large number of species are to be compared. As a possible solution, the use of nymphs, alone or in combination with adult samples, could be a route to optimize statistical performance.

The Cimicidae species examined in our study are macroscopically similar, and expertise is required for their identification. Similarities in appearance are a well-documented aspect of the Cimicidae family in general [9]. They were thus selected as interesting subject material to explore the usefulness of the taxonomic signal of the pronotum. The presence of *C. hemipterus* in a train station in Bangkok suggests that bed bugs have extended their habitat throughout the country, indicating a need to expand surveillance efforts in preparation for improved control, management, and insecticide resistance [50]. The identification of *S. parvus* as the species collected in Thap Kwang, Kaeng Khoi, Saraburi Province of Thailand, is not surprising—the species had been already discovered in that area and was suspected to be the vector of Kaeng Khoi virus transmission from bat hosts to mine workers [6]. Bats are natural reservoir hosts for multiple arboviruses associated with human disease [51]. A repeated exposure to bat bugs could lead to human transmission of zoonotic viruses, as was suggested for the case of Kaeng Khoi virus (KKV) [6]. *Paracimex* sp. was collected by Chittsamart et al., 2015 [35], from an edible-nest swiftlets’ cave during an investigation into an island population of sand flies on Lang Ga Jiew Island of Chumphon Province, Thailand. The ectoparasite was collected among others for the attempts of determining the species responsible for the frequent bug bites experienced by bird’s nest collectors and guards in the cave’s surrounding environment. However, no evidence is yet available to implicate *Paracimex* sp. Investigating this will help determine the potential transmission of zoonotic diseases to humans by cave-adapted species [52,53].

## 5. Conclusions and Recommendations

Our study has provided evidence that, to distinguish among three species of Cimicidae, (i) it is feasible to use only one anatomical structure, and (ii) outline-based morphometry is the most applicable method. The geometric approach not only reliably captured the shape, but it also provided an efficient estimate of the pronotum global size, which probably reflects the total size of the specimen.

The implementation of outline-based morphometry for routine taxonomic work on a few specimens requires the existence of pre-established reference data, which is also the case for molecular data. Such reference data could be a bank of images showing insects (or, at least, their pronotum) belonging to various species, which could allow one to identify a single field specimen using only morphometrics [54]. The ability of the outline-based method to discriminate among three taxa, two of which are medically important genera (*C. hemipterus* and *S. parvus*), suggests it could be used as a new tool for infestation monitoring. In addition, it is not time-consuming and does not require highly specialized skills. We thus propose that the viability of the method be tested with a much larger number of species.

## Figures and Tables

**Table 1:** Average values of log-size variables and semi-major axis for both sexes and per species from the linear and outline-based morphometry, respectively.

Species	Linear Morphometry		Outline-Based Morphometry	
	Male	Female	Male	Female
*Paracimex* sp.	0.44 ± 0.02 ^a^ (*n* = 14)	0.43 ± 0.03^a^ (*n* = 12)	0.70 ± 0.05 ^a^ (*n* = 14)	0.70 ± 0.05 ^a^ (*n* = 12)
*Cimex hemipterus*	0.37 ± 0.12 ^a^ (*n* = 7)	0.45 ± 0.04 ^a,^* (*n* = 19)	0.90 ± 0.24 ^a^ (*n* = 7)	1.32 ± 0.14 ^b,^* (*n* = 19)
*Stricticimex parvus*	0.30 ± 0.01 ^b^ (*n* = 12)	0.30 ± 0.02 ^b^ (*n* = 14)	0.40 ± 0.07 ^b^ (*n* = 12)	0.30 ± 0.04 ^c,^* (*n* = 14)

^a,b,c^ Significant differences between species at *p* < 0.05. * Significant differences between male and female among species at *p* < 0.05. *n*: number of samples.

**Table 2:** Comparison of classification scores based on size and shape for both sexes from traditional morphometry and outline-based morphometry. Total performance is presented as a percentage of assigned/observed.

Validated Reclassification Scores	Linear Morphometry		Outline-Based Morphometry	
	Male	Female	Male	Female
Maximum likelihood size-based reclassification score	61% (20/33)	71% (32/45)	85% (28/33)	82% (37/45)
Multilayer perceptron shape-based reclassification score	99% ± 0.7%	94% ± 0.5%	96% ± 0.5%	98% ± 0.7%

**Table 3:** Estimated influence of size variation on shape variables in males and females (the allometric effect) for linear and outline-based morphometry.

	Males	Females
	Linear morphometry	
PC1	91%	91%
PC2	8%	5%
	Outline-based morphometry	
PC1	15%	2%
PC2	66%	37%

## Data Availability

Due to ownership reasons, the data presented in this study are available on request from the corresponding author.

## Supplementary Materials

The following supporting information can be downloaded at: https://www.mdpi.com/article/10.3390/insects13121155/s1; Table S1: The samples used in this study, including collection area, year of collection, and number of specimens per species; Table S2: Pronotum features generally used for morphological analysis of *Cimex* spp. and other genera and species in the literature [12,13,23,47]. Measure of the pronotum features (mean ± SD; mm) obtained using an ocular micrometer for linear morphometric analysis. (References [12,13,23,47] are cited in both the main text and Supplementary Materials).
