# Supplementary material for: Comparison of Two Different Morphological Methods to Study the Pronotum of Cimicidae: Bed Bugs, Bat Bugs, and Swallow Bugs
Source: Insects. 2022 Dec 14;13(12):1155. doi: 10.3390/insects13121155 (PMC9785296; doi:10.3390/insects13121155)
Supplement: Supplementary file 1 [file insects-13-01155-s001.zip › insects-2075701-supplementary.pdf]

Table S1: The samples used in this study, including their collection area, the year of collection, and the number of specimens used per species.

Samples	Sex	Locality	Longitude	Latitude	Year of collection	Host
Stricticimex parvus	12 males, 14 females	Saraburi Province	14° 36' 6.5412'' N	101° 7' 20.7372'' E	2014	Bat
Cimex hemipterus	7 males, 19 females	Bangkok Province	13° 44' 22.5" N	100°30' 58.4" E	2014	Human
Paracimex sp.	14 males, 12 females	Chumphon province	10° 19' 06.9" N	99°18' 03.9" E	2013	Bird

Table S2: Pronotum features generally used for morphological analysis of *Cimex* spp. and for other genera and species in the literature [12,13,23,47]. Measure of the pronotum features (mean ± SD; mm) obtained from the ocular micrometer for the linear morphometric analysis.

**Features**	**Used for genus *Cimex***	**Used for genus and species distinction**	*Paracimex avium*		*Cimex hemipterus*		*Stricticimex parvus*	
			**Male (n=14)**	**Female (n=12)**	**Male (n=7)**	**Female (n=19)**	**Male (n=12)**	**Female (n=14)**
Pronotum width (pw)	x	x	1.24 ± 0.08	1.25 ± 0.10	1.27 ± 0.36	1.50 ± 0.21	0.66 ± 0.03	0.67 ± 0.04
Depth of the anterior pronotal concavity (pc)	x	x	0.02 ± 0.003	0.02 ± 0.003	0..01 ± 0.004	0.02 ± 0.02	0.003 ± 0.001	0.003 ± 0.001
Pronotum length (medial) (pm)	x	-	0.63 ± 0.06	0.61 ± 0.07	0.38 ± 0.24	0.51 ± 0.08	0.36 ± 0.03	0.38 ± 0.04

n: number of samples
x: used
-: not used
